# Selective Activation of KCa3.1 and CRAC Channels by P_2_Y_2_ Receptors Promotes Ca^2+^ Signaling, Store Refilling and Migration of Rat Microglial Cells

**DOI:** 10.1371/journal.pone.0062345

**Published:** 2013-04-19

**Authors:** Roger Ferreira, Lyanne C. Schlichter

**Affiliations:** 1 Genes and Development Division, Toronto Western Research Institute, University Health Network, Toronto, Ontario, Canada; 2 Department of Physiology, University of Toronto, Toronto, Ontario, Canada; Brigham & Women's Hospital-Harvard Medical School, United States of America

## Abstract

Microglial activation involves Ca^2+^ signaling, and numerous receptors can evoke elevation of intracellular Ca^2+^. ATP released from damaged brain cells can activate ionotropic and metabotropic purinergic receptors, and act as a chemoattractant for microglia. Metabotropic P_2_Y receptors evoke a Ca^2+^ rise through release from intracellular Ca^2+^ stores and store-operated Ca^2+^ entry, and some have been implicated in microglial migration. This Ca^2+^ rise is expected to activate small-conductance Ca^2+^-dependent K^+^ (SK) channels, if present. We previously found that SK3 (KCa2.3) and KCa3.1 (SK4/IK1) are expressed in rat microglia and contribute to LPS-mediated activation and neurotoxicity. However, neither current has been studied by elevating Ca^2+^ during whole-cell recordings. We hypothesized that, rather than responding only to Ca^2+^, each channel type might be coupled to different receptor-mediated pathways. Here, our objective was to determine whether the channels are differentially activated by P_2_Y receptors, and, if so, whether they play differing roles. We used primary rat microglia and a rat microglial cell line (MLS-9) in which riluzole robustly activates both SK3 and KCa3.1 currents. Using electrophysiological, Ca^2+^ imaging and pharmacological approaches, we show selective functional coupling of KCa3.1 to UTP-mediated P_2_Y_2_ receptor activation. KCa3.1 current is activated by Ca^2+^ entry through Ca^2+^-release-activated Ca^2+^ (CRAC/Orai1) channels, and both CRAC/Orai1 and KCa3.1 channels facilitate refilling of Ca^2+^ stores. The Ca^2+^ dependence of KCa3.1 channel activation was skewed to abnormally high concentrations, and we present evidence for a close physical association of the two channel types. Finally, migration of primary rat microglia was stimulated by UTP and inhibited by blocking either KCa3.1 or CRAC/Orai1 channels. This is the first report of selective coupling of one type of SK channel to purinergic stimulation of microglia, transactivation of KCa3.1 channels by CRAC/Orai1, and coordinated roles for both channels in store refilling, Ca^2+^ signaling and microglial migration.

## Introduction

In the mature CNS, microglial cells with highly branched processes continually survey the local microenvironment and rapidly respond to stranger and danger signals [1]. Migration to the site of damage is an essential component of the microglial response to acute CNS injury. ATP, which is released from damaged cells, can bind to microglial ionotropic (P_2_X) and metabotropic (P_2_Y) purinergic receptors and promote migration [2]–[4]. *In vitro* studies on microglia migration have focused on the roles of P_2_X_4_ and P_2_Y_12_ [5]–[7]. Microglial P_2_Y receptors rapidly elevate intracellular free Ca^2+^ by coupling Ca^2+^ release from stores to store operated Ca^2+^ entry (SOCE) [8], [9]. Thus, it is expected that P_2_Y receptors will link extracellular damage signals to intracellular Ca^2+^, microglial activation and migration. The SOCE pathway used by microglia for migration following P_2_Y receptor activation has not been identified. By combining molecular, biophysical and pharmacological approaches, we previously identified the Ca^2+^-release activated Ca^2+^ (CRAC) channel as a major SOCE pathway in primary rat microglia [10]. More recently, we discovered a contribution of CRAC channels to microglial migration and the formation of podosomes [11].

An expected immediate response to elevated intracellular Ca^2+^ in microglia is opening of SK (small-conductance Ca^2+^-activated K^+^) channels. We previously showed that SK4 (KCa3.1) [12] and SK3 (KCa2.3) channels [13] are expressed in rat microglia, and regulate activation evoked by lipopolysaccharide; i.e., p38MAPK activation, iNOS up-regulation and nitric oxide production, and the ability of microglia to kill neurons. At the time, we hypothesized that the SK channels contribute to microglial activation by maintaining a negative membrane potential and thus, a large driving force for Ca^2+^ influx through CRAC channels. However, the SK currents were not monitored, and roles of SK3 and KCa3.1 channels in regulating SOCE have not been examined in microglia. Recently, we discovered that both SK3 and KCa3.1 currents are reliably activated in the MLS-9 microglia cell line by the neuroprotective drug, riluzole, with little or no rise in intracellular Ca^2+^ [14]. This finding contradicts the prevailing view that riluzole simply sensitizes SK channels so that they open at resting Ca^2+^ levels [15]. Furthermore, neither SK3 nor KCa3.1 current was activated simply by raising Ca^2+^ to ∼1 µM, which is well above the normal EC_50_ values reported for native and heterologously expressed channels (see Discussion). Instead, our results on MLS-9 cells raise the possibility that SK3 and KCa3.1 channels in microglia require more than a simple elevation in Ca^2+^. If so, it is possible that the two channel types can selectively respond to different stimuli.

This study was designed to address three overall questions. First, we asked whether metabotropic P_2_Y_2_ receptors in microglia elevate intracellular Ca^2+^ and activate SK3 and KCa3.1 channels, and if so, whether this requires Ca^2+^ entry through CRAC channels. Having found that only KCa3.1 channels were activated, and that CRAC channels were involved, we next asked whether KCa3.1 channels selectively control store-operated Ca^2+^ entry and store refilling. This was the case. Finally, we asked whether P_2_Y_2_ receptors increase microglia migration through mechanisms that require CRAC and the selective activation of KCa3.1. Again, we found this to be the case.

## Materials and Methods

### Cells

#### Ethics statement

Animals were used in strict accordance with the guidelines established by the Canadian Council on Animal Care, and was approved by the Animal Care Committee of the University Health Network (AUP #914). Primary cultures of rat microglia (≥98% pure) were prepared from brains harvested from 1–2 day old Sprague Dawley pups (Charles River, St-Constant, Quebec, Canada) Essentially pure microglia cultures were prepared according to our standard protocols [10], [12], [13], [16]. That is, following removal of the meninges, the brain was minced in cold Minimal Essential Medium (MEM; Invitrogen, Burlington, ON, Canada). The dissociated tissue was centrifuged (300×g, 10 min) and re-suspended in MEM supplemented with 10% fetal bovine serum (FBS) (from Wisent, St-Bruno, PQ), and 0.05 mg/mL gentamycin (Invitrogen). After two days growth in tissue culture flasks, the supernatant containing cellular debris and non-adherent cells was removed and replaced with fresh medium. The mixed cell cultures were allowed to grow for another 8 days, and were then shaken on an orbital shaker (65 rpm, 3–4 h, 37°C, 5% CO_2_). The resulting suspension of non-adherent microglia was centrifuged (300×g, 10 min), the pellet was re-suspended in MEM with reduced serum (2% FBS). Under these conditions, we have found that the microglial cells are a relatively non-activated state [16].

Our laboratory derived the MLS-9 cell line many years ago by treating pure cultures of rat microglia with colony stimulating factor-1 for several weeks. Individual cell colonies were harvested and used to establish continuous cell lines, of which we named one, MLS-9 [17]. We have used this cell line extensively for studies of K^+^ channels [14], [17]–[20] and Cl^−^ channels [21]. MLS-9 cells were thawed and cultured for several days in culture medium (MEM, 10% FBS, 100 µM gentamycin), and then harvested in phosphate buffered saline (PBS) containing 0.25% trypsin and 1 mM EDTA, washed with MEM, centrifuged (300×g, 10 min) and re-suspended in culture medium. MLS-9 cells were plated in the culture medium at 4.5×10^4^ cells/coverslip for Ca^2+^ imaging and patch-clamp analysis. An important advantage of using MLS-9 cells is that they lack three currents that can interfere with isolating Ca^2+^-activated K^+^ currents. Primary rat microglia have an inward-rectifier K^+^ current at negative membrane potentials [22], [23], a large outward Kv1.3 current that activates above about −30 mV [22], and TRPM7, which produces a large current at positive potentials [24].

Chemicals were from Sigma-Aldrich, unless otherwise indicated. Stock solutions of several antagonists were made with DMSO; i.e., the P_2_Y receptor blocker, suramin, the Orai1/CRAC blockers 2-APB and BTP2, and the KCa3.1 blocker, TRAM-34. Uridine 5′-triphosphate (trisodium salt dehydrate) and the SK1-SK3 blocker, apamin, were dissolved in double distilled water. All stock solutions were aliquoted and stored at –20°C until used.

### Intracellular free Ca^2+^

The Fura-2 imaging methods were the same as we recently described [14]. MLS-9 cells growing on glass coverslips (∼5×10^4^ cells per 15 mm diameter coverslip) were incubated at room temperature with 3.5 µg/ml Fura-2AM (Invitrogen) for 45 min in the dark. A coverslip was then mounted in a 300 µl volume perfusion chamber (Model RC-25, Warner Instruments, Hamden CT), containing the same bath solution as for patch-clamping (see below). The effects of ion channel blockers (50 µM 2-APB, 10 µM BTP2, 1 µM TRAM-34, 100 nM apamin) on UTP-evoked calcium signals were assessed on different batches of cells from separate coverslips. Images were acquired at room temperature using a Nikon Diaphot inverted microscope, Retiga-EX camera (Q-Imaging, Burnaby, BC, Canada), and Northern Eclipse image acquisition software (Empix Imaging, Mississauga, ON, Canada). A Lambda DG-4 Ultra High Speed Wavelength Switcher (Sutter Instruments, Novato, CA) was used to alternately acquire images at 340 and 380 nm excitation wavelengths. Images were acquired every 4 s, and the excitation shutter was closed between acquisitions to prevent photobleaching. The intracellular free Ca^2+^ concentration was calculated from the standard equation [25] as before [14]. For every experiment, cells on a matched coverslip (i.e., not exposed to UTP) were exposed to 2 µM ionomycin to obtain the maximum 340∶380 ratio with saturating calcium. Then, a Ca^2+^-free bath solution with 2 µM ionomycin and 3 mM MnCl_2_ was perfused in to obtain the minimum 340∶380 ratio.

### Patch-clamp electrophysiology

MLS-9 cells were plated on 15 mm diameter coverslips (4.5×10^4^/coverslip), mounted in the same perfusion chamber as for Ca^2+^ imaging. The cells were superfused with an extracellular (bath) solution containing the following (in mM): 125 NaCl, 5 KCl, 1 MgCl_2_, 1 CaCl_2_, 5 glucose, and 10 HEPES, adjusted to pH 7.4 (with NaOH) and to ∼300 mOsm with sucrose. For Ca^2+^-free bath solution, CaCl_2_ was omitted and 1 mM EGTA was added. Bath solutions were exchanged using a gravity-driven perfusion system flowing at 1.5–2 ml/min and all recordings were made at room temperature. Whole-cell recordings were made with pipettes pulled from thin-walled borosilicate glass (WPI, Sarasota, FL) to a resistance of 5–8 MΩ using a Narishige puller (Narishige Scientific, Setagaya-Ku, Tokyo). Pipettes were filled with a solution (intracellular) containing (in mM): 100 K-aspartate, 40 KCl, 1 MgCl_2_, 2 MgATP, and 10 HEPES, pH adjusted to 7.2 (with KOH), 280 mOsm/kgH_2_O. Either 1 EGTA+0.5 CaCl_2_ or 1 BAPTA+0.45 CaCl_2_ (where noted) was used to buffer the initial internal free Ca^2+^ to ∼120 nM (calculated using WEBMAXC Extended software, http://www.stanford.edu/~cpatton/webmaxc/webmaxcE.htm). We chose low buffer concentrations (1 mM) to allow the purinergic receptor agonist, UTP, to transiently elevate intracellular Ca^2+^. Recordings were made with an Axon Multiclamp 700A amplifier, compensated on-line for capacitance and series resistance. Patch-clamp data were filtered at 5 kHz, and acquired and digitized using a Digidata 1322A board with pClamp software. Junction potentials were reduced by using agar bridges made with bath solution, and were calculated with the utility in pCLAMP. After correction, all reported voltages were about 5 mV more negative than reported in the text and figures.

### Immunocytochemistry

Standard methods were used, similar to our recent papers [11], [13], [26]. In brief, antibodies were diluted in 4% donkey serum and centrifuged (8200×g, 10 min) to precipitate any aggregated antibody. MLS-9 cells were fixed for 10 min in 4% paraformaldehyde and washed (3×, 5 min each). This was followed by antigen retrieval in hot citrate buffer, which we found necessary for optimal Orai1 staining [11]. Cells were washed and permeabilized for 5 min with 0.2% Triton X-100, and washed in PBS (3×, 5 min each). Non-specific antigens were blocked with 4% donkey serum for 1 h at room temperature. The cells were then incubated with the following primary antibodies overnight at 4°C: anti-Orai1 (goat polyclonal, 1∶100; Santa Cruz Biotechnology; Santa Cruz, CA), and anti-KCa3.1 (SK4) (rabbit polyclonal, anti-serum, 1∶1000; Abcam; Cambridge, MA). The cells were washed in PBS (4×, 5 min each), followed by another block with 4% donkey serum and 0.01% BSA for 1 h at room temperature. The cells were then incubated (1 h, room temperature) with the secondary antibodies: Alexa Fluor 488-conjugated (green) bovine anti-goat (1∶1000; Jackson Immunoresearch; West Grove, PA, USA), and DyLight 594-conjugated (red) donkey anti-rabbit (1∶250; Jackson Immunoresearch; West Grove, PA, USA). Negative controls were prepared using the same protocol, but omitting each primary antibody. After washing in PBS (4×, 5 min each) cell nuclei were labelled for 5 min with DAPI (4′-6-diamidino-2-phenylindole) (1∶3000; Sigma-Aldrich), and then washed in PBS (3×, 5 min each). The coverslips were mounted on glass slides with DAKO mounting medium (Dako; Glostrup, Denmark). Cells were imaged with an Axioplan 2 microscope using a 63× objective lens. Images were recorded with an Axiocam HRm digital camera, deconvolved and analyzed with Axiovision 4.6 software (all from Zeiss; Toronto, ON).

### Transwell^TM^ migration assay

Cultured primary rat microglia (2–3×10^4^ cells/insert) were seeded on the upper inserts of 24-well Transwell^TM^ Migration Chambers (VWR, Mississauga, ON), which contained uncoated filters with open 8 µm-diameter holes. Microglia were bathed in MEM with 2% FBS and allowed to adhere for ∼1 h prior to adding compounds. When used, UTP was added to the lower well to act as a chemoattractant, and each antagonist was added to the upper well. The chamber was incubated (24 h, 37°C, 5% CO_2_). To quantify the transmigration of microglia to the underside of each filter, we first removed the remaining cells from the upper side, using a Q-tip^TM^. The filters were fixed (4% PFA, 15 min), rinsed 3× in PBS, stained with 0.5% crystal violet (1 min), quickly rinsed, and allowed to air dry. Microglia that had migrated to the underside of each filter were viewed at 20× with an Olympus CK2 Phase Contrast Inverted microscope (Olympus, Tokyo, Japan). Cells were counted in 5 fields of view per filter.

### Statistical analysis

All data are expressed as mean ± SEM. For analysis of drug effects on currents, Ca^2+^ signaling, and microglia migration, 1-way ANOVA and Tukey's post-hoc tests for multiple comparisons were conducted using GraphPad Prism ver 5.01 (GraphPad Software, San Diego, CA). Values of *p*<0.05 were taken as statistically significant.

## Results

### UTP activates a KCa3.1 current: Requirement for high intracellular Ca^2+^

**Figure 1:**
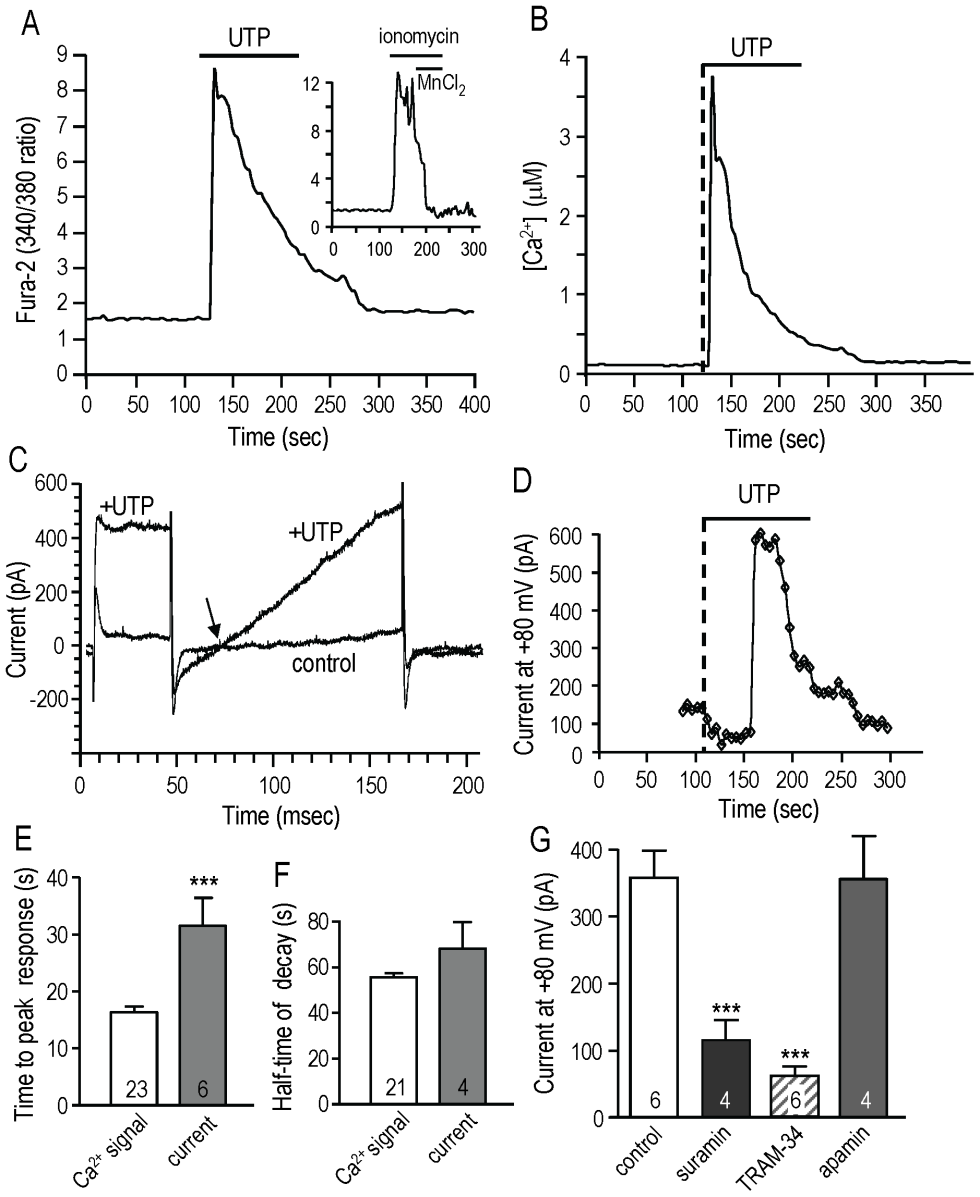
The metabotropic purinergic receptor agonist, UTP, elevates Ca^2+^ elevation and selectively activates a KCa3.1 current. **A.** A representative Fura-2 recording from an MLS-9 cell (rat microglia cell line), in which 100 µM UTP was bath applied during the period marked by the horizontal bar. The inset shows how the Ca^2+^ signal was calibrated by adding ionomycin and MnCl_2_, and as described in the Methods. **B.** The time course of the calibrated UTP-evoked Ca^2+^ rise (in µM) is shown for the cell in panel A. **C.** In whole-cell patch-clamp recordings, UTP evoked a large, transient current. From a holding potential of −70 mV, a voltage step to +50 mV was followed by a ramp from −100 to +80 mV applied every 5 sec. The two traces show the baseline control current (standard bath solution) and at the peak after adding 100 µM UTP. The reversal potential is indicated by the arrow. **D.** The time course of the current measured at +80 mV (same cell as panel C) is plotted on the same time scale as the Ca^2+^ signal in panel B. Vertical dashed lines indicate the time at which UTP was added. **E.** Comparison of the mean time to peak response after adding UTP for the Ca^2+^ signal and current activation. **F.** Comparison of the time course (half time) of the decay phase of the Ca^2+^ signal and current. **G.** UTP selectively activates a KCa3.1 current. The UTP-dependent current was quantified after subtracting the baseline from the maximal current (both at +80 mV) in the absence (control) or presence of the P_2_Y_2_ and P_2_Y_6_ receptor antagonist, 100 µM suramin. UTP-evoked currents were recorded in separate cells with the selective KCa3.1 blocker (1 µM TRAM-34) or the SK1–SK3 blocker (100 nM apamin) in the bath. The number of cells is indicated on each bar. ***p<0.001

The metabotropic purinergic receptor agonist, UTP, induced a biphasic Ca^2+^ rise, with an initial rapid increase and a more slowly decaying phase (Fig. 1A). The Ca^2+^ concentration was calculated by determining the maximal and minimal 340/380 values (example shown in inset, and see Methods) for each cell. For this cell, Ca^2+^ rapidly increased to ∼4 µM, spontaneously declined, and then returned to baseline after UTP was washed out (Fig. 1B). On average, the peak Ca^2+^ level was 5.3±1.2 µM (n = 19). After UTP was washed out, Ca^2+^ decreased to ∼85 nM, which is similar to our previous results for MLS-9 cells [14].

The MLS-9 microglia cell line has several of the same ion channels as primary rat microglia, and we have extensively used these cells. MLS-9 cells have important advantages for studying small-conductance Ca^2+^-activated K^+^ channels. Both SK3 (KCa2.3) and KCa3.1 currents are reliably activated (by riluzole) [14], and we have not detected voltage-gated or TRPM7 currents, which complicate the recordings from primary rat microglia. In the present study, the first surprising finding was that UTP selectively activated a large KCa3.1 current, which was identified as follows. UTP activated a current in all cells tested (n>30). The outward current showed no time dependence during voltage-clamp steps (Fig. 1C), and thus current was seen at all voltages tested with the ramp protocol (−100 to +80 mV). The reversal potential was about −75 mV (−80 mV after junction potential correction), which is very close to the K^+^ Nernst potential (−85 mV) with the bath and pipette solutions used. The UTP-evoked K^+^ current was transient (Fig. 1D), and its peak was delayed (32±5 s; n = 6) compared with the rapid Ca^2+^ rise (16±1 s, n = 23) (Fig. 1E). Instead, the decay phase of the current temporally matched the decay of the Ca^2+^ signal. The half-time (t_½_) for the Ca^2+^ decay was 56±2 s (n = 21) and 68±12 s (n = 4) for the current (Fig. 1F). This temporal relationship suggests that, rather than the initial Ca^2+^ rise, it is the secondary phase of the Ca^2+^ signal that is responsible for activating KCa3.1. The pharmacological data (Fig. 1G) show that the amplitude (358±41 pA at +80 mV; n = 6) was reduced to 116±30 pA (n = 4) by the P_2_Y_2_/P_2_Y_6_ receptor antagonist, suramin [27], and to 60.2±14.1 pA by the KCa3.1-selective blocker, TRAM-34 (n = 6). The SK1–SK3 blocker, apamin, had no effect. Thus, we conclude that UTP selectively activated KCa3.1 current. The pharmacology—activation by 100 µM UTP, inhibition by 100 µM suramin—implicates the P_2_Y_2_ receptor (see Discussion) in trans-activating KCa3.1.

**Figure 2:**
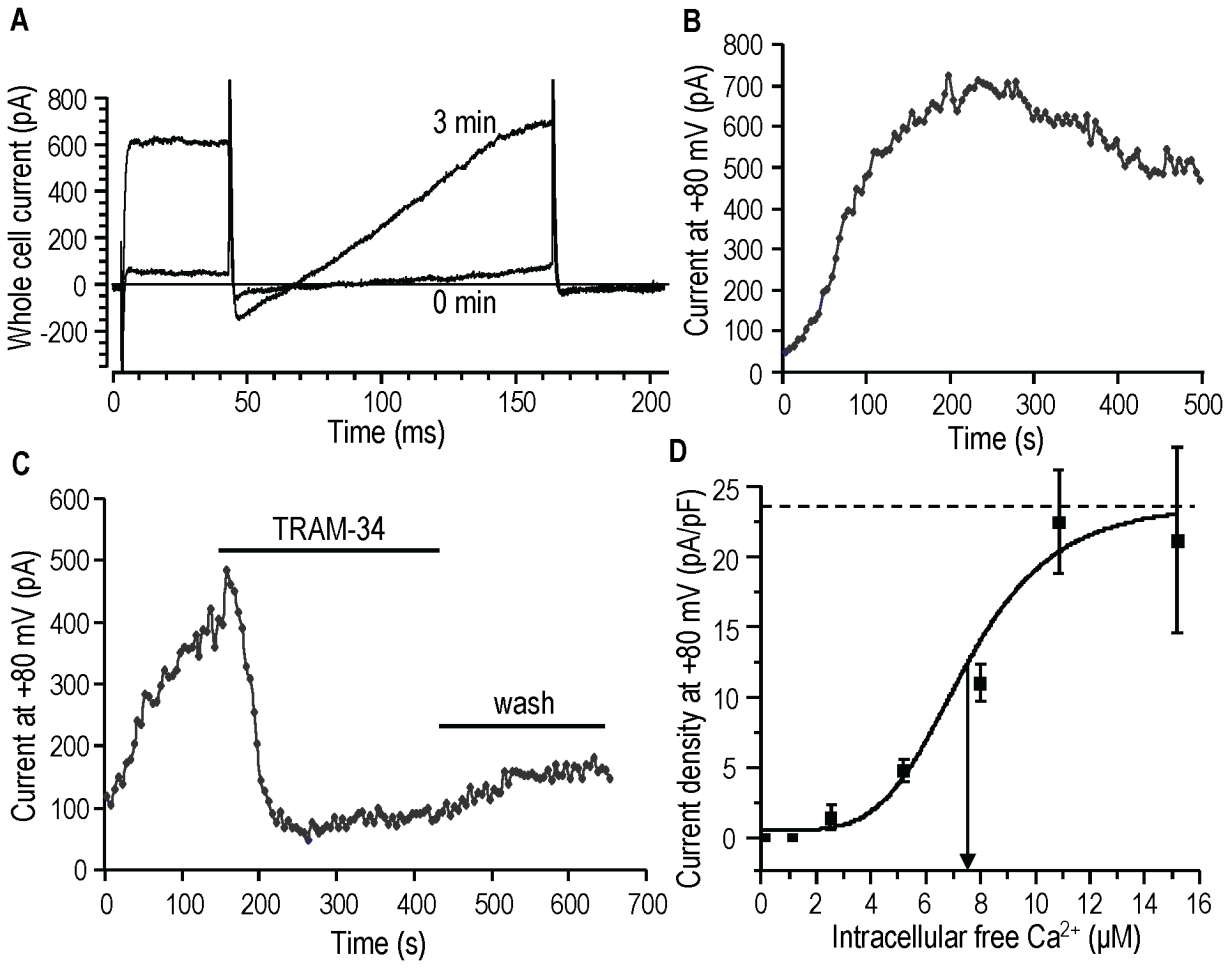
KCa3.1 current activation requires unexpectedly high intracellular free Ca^2+^. For these experiments on MLS-9 cells, the bath contained 100 nM apamin to eliminate any possible contribution of SK3. **A.** The two traces (same voltage protocol as in Fig 1) show a small current immediately after establishing the whole-cell recording (0 min) and 3 min later. The pipette solution contained 10.9 µM free Ca^2+^. **B, C.** The time course of the current measured at +80 mV is shown for two cells with 10.9 µM free intracellular Ca^2+^. The cell in panel A is shown in B. For a different cell (panel C), the KCa3.1 blocker, 1 µM TRAM-34, was added to the bath after the current reached a plateau, and then washed out with standard bath solution. **D.** Ca^2+^-dependence of KCa3.1 current activation. The current density (pA/pF) as a function of intracellular free Ca^2+^ is fitted to the Hill equation. The maximal current density is indicated by the dashed line, and the EC_50_ is indicated by the vertical arrow.

The peak of the UTP-response reached a high Ca^2+^ level (5.3 µM); therefore, we next examined the Ca^2+^-dependence of current activation without UTP. Whole-cell recordings were established with pipette solutions containing 2.5, 5.2, 8.0, 10.9 or 15.3 µM free Ca^2+^. The bath contained apamin to preclude any possible contribution of SK3. The example cell shows activation of KCa3.1 current with 10.9 µM intracellular Ca^2+^ (Fig. 2A), a gradual increase to a peak at ∼3 min and a quasi-stable plateau (Fig. 2B). The current was KCa3.1, as shown by the rapid block by TRAM-34 in a separate cell (Fig. 2C). A Ca^2+^ dose-response curve was constructed by plotting current density (pA/pF) versus intracellular free Ca^2+^ (Fig. 2D). It includes our previous data with 1.1 µM (n = 10 [14]) and 0.1 µM Ca^2+^ (n>100 [14], and unpublished). From this relationship, the maximal current density was 23.6±2.9 pA/pF, the EC_50_ was 7.6±0.7 µM, and the Hill coefficient was 4.6±1.7. Similar EC_50_ and Hill coefficient values were obtained using current density or slope conductance. Surprisingly, this EC_50_ is well above the previously reported sub-micromolar values for heterologously expressed KCa3.1 channels (see Discussion), and there was no current at 1.1 µM Ca^2+^.

### KCa3.1 requires store-operated Ca^2+^ entry and promotes store refilling

**Figure 3:**
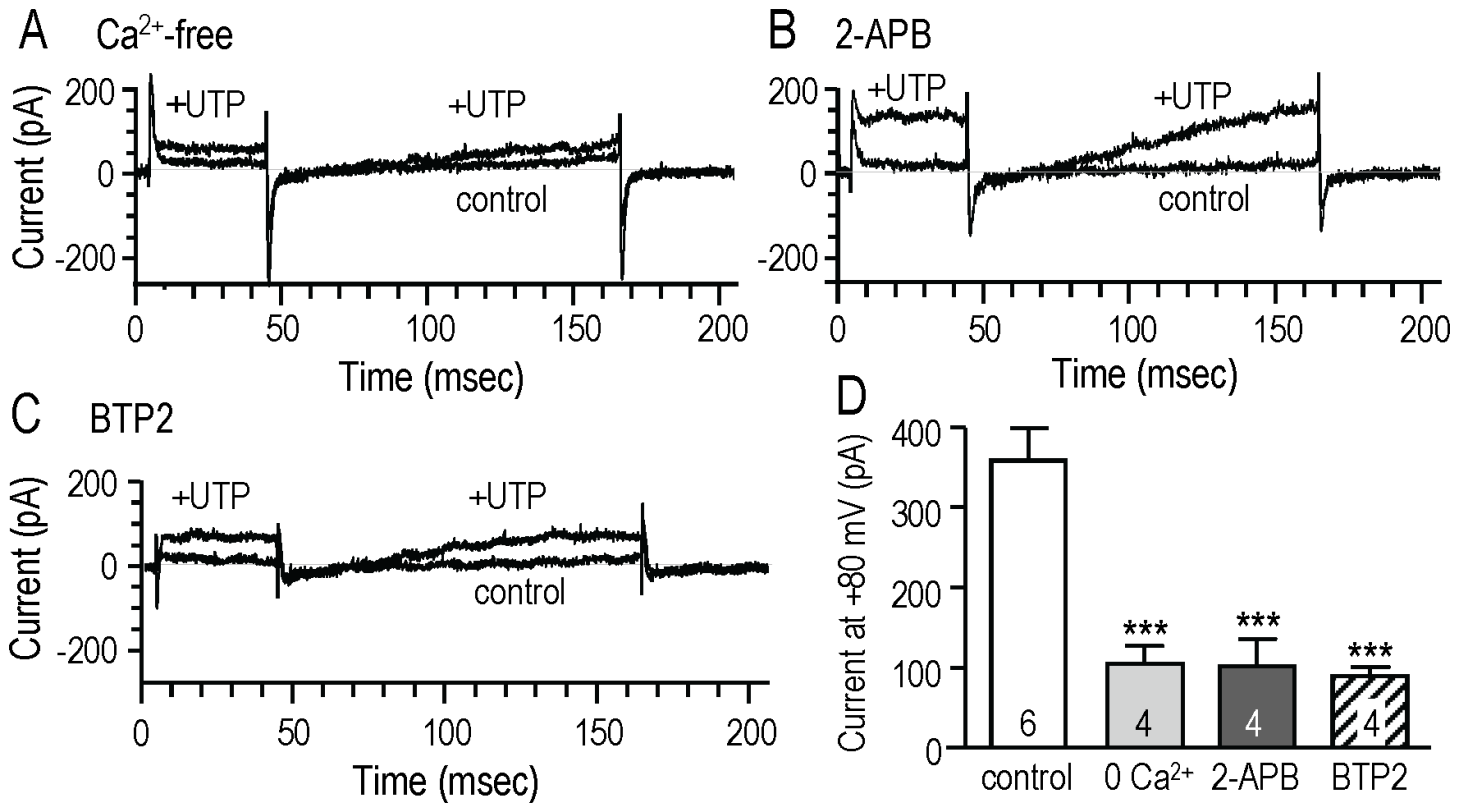
The UTP-evoked KCa3.1 current activation involves Ca^2+^ entry and Ca^2+^-release activated Ca^2+^ (CRAC) channels. The current was evoked by UTP in separate MLS-9 cells with Ca^2+^-free bath solution (**A**), or with either 50 µM 2-APB (**B**) or 10 µM BTP2 (**C**) in the bath. **D.** The amplitude was quantified (as in Fig. 1E) and compared with standard bath solution. The number of cells is indicated on each bar. ***p<0.001

In microglia, as in other cell types, activation of metabotropic purinergic receptors causes IP_3_-mediated release of Ca^2+^ from intracellular stores, followed by store-operated Ca^2+^ entry and store refilling [8]. In the MLS-9 microglia cell line, UTP evoked a P_2_Y receptor-mediated biphasic rise in Ca^2+^ (Fig. 1A,B) and activated a KCa3.1 current (Figs. 1C–E, Fig. 2). To assess whether the transient KCa3.1 activation requires Ca^2+^ influx, we compared the UTP-evoked current with and without Ca^2+^ in the bath (Fig. 3A). The summarized data (Fig. 3D) show that removing external Ca^2+^ decreased the current amplitude by 71%, from 358±41 pA (n = 6, same cells as in Fig. 1E) to 104±23 pA (n = 4). This demonstrates a need for Ca^2+^ influx. We previously showed that store-operated Ca^2+^ entry into rat microglia involves currents mediated by the Ca^2+^-release activated Ca^2+^ (CRAC) channel [10], whose pore-forming subunit is Orai1 [28]. To analyze whether Ca^2+^ influx through CRAC channels is needed to activate KCa3.1 current, we used 50 µM 2-APB (Fig. 3B, D), a concentration that blocks CRAC/Orai1 channels [29], and the more selective CRAC/Orai1 blocker, 10 µM BTP2 [30], [31] (Fig. 3C, D). The KCa3.1 current was greatly reduced by both blockers; i.e., by 72% with 2-APB (to 102±34 pA, n = 4) and by 75% with BTP2 (to 90±11 pA, n = 4). Together, these results implicate Ca^2+^ influx through CRAC channels in activating KCa3.1 channels after P_2_Y receptor stimulation.

**Figure 4:**
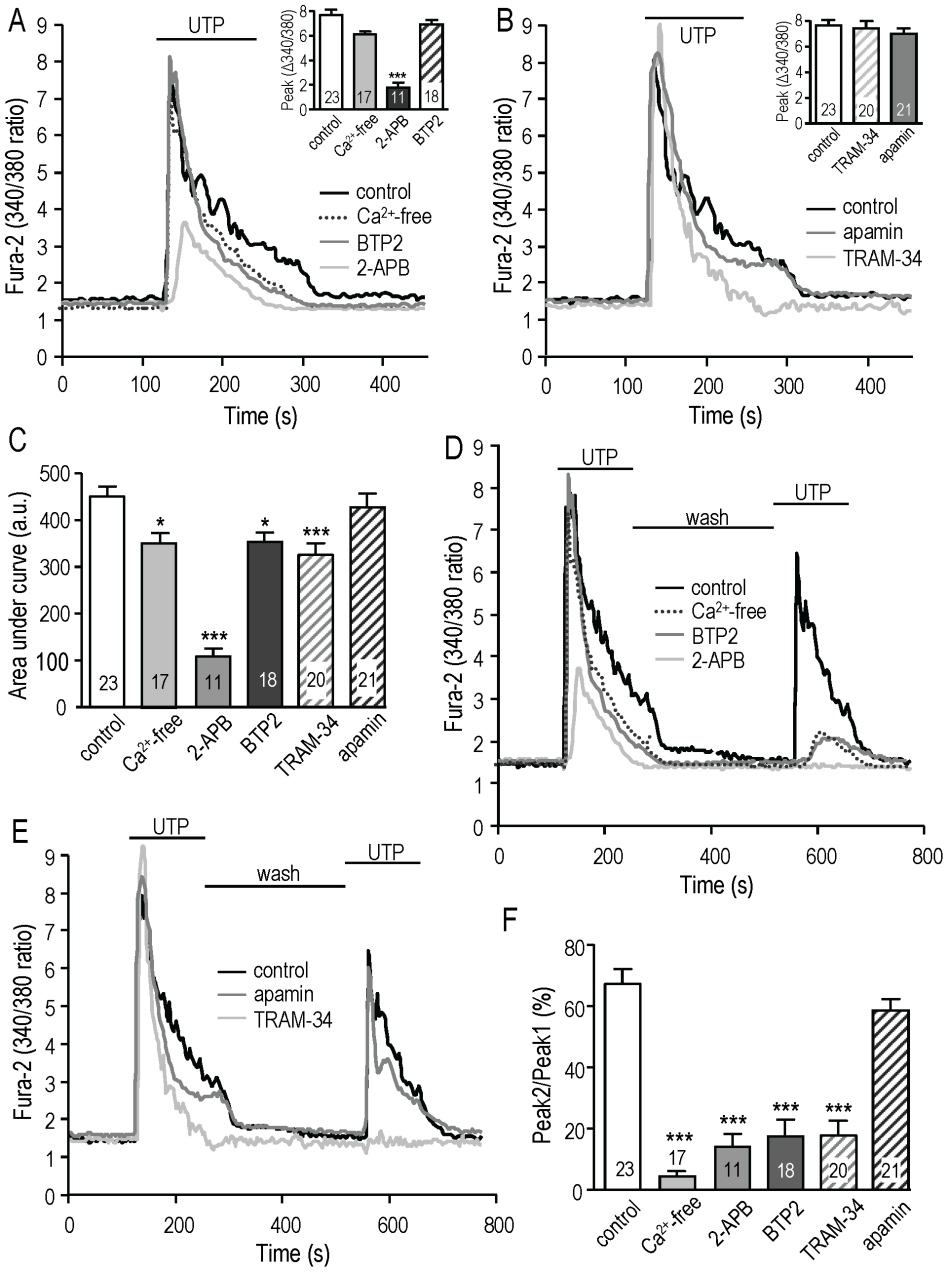
KCa3.1 activation promotes store-operated Ca^2+^ entry and refilling, and involves CRAC channels. When used, the Ca^2+^-free solution or ion channel blocker (50 µM 2-APB, 10 µM BTP2, 1 µM TRAM-34, 100 nM apamin) was present in the bath throughout the recording period. **A, B.** Representative Fura-2 traces show the UTP-evoked Ca^2+^ signals from six MLS-9 cells on six separate coverslips. The same control cell is shown in A and B. The insets show the summarized peak responses for the number of cells indicated on each bar. ***p<0.001 **C.** Summary of the area under the curve (arbitrary units) calculated for the first 4 min of the response (same treatments as in A and B). **D, E.** Representative Fura-2 traces showing the first and second responses to UTP, with a 5-min recovery period between stimuli. The same control cell is shown in D and E. **F.** Summary comparing the maximal responses to the first and second UTP stimulus; i.e., Peak 2/Peak 1×100 (same treatments as in D and E). In panels C and F, the number of cells is indicated on each bar, and *p<0.05, **p<0.01, ***p<0.001

We next asked whether CRAC and KCa3.1 channels contribute to the Ca^2+^ rise following P_2_Y receptor activation with UTP. None of the channel blockers affected the baseline Ca^2+^ level. When Ca^2+^ was omitted from the bath, UTP evoked only a rapidly rising Ca^2+^ transient due to release from internal stores and its peak amplitude was unchanged (Fig. 4A). The same response was seen with the blocker, 10 µM BTP2, which selectively blocks Ca^2+^ entry through CRAC channels. The secondary decay phase of the Ca^2+^ response was decreased by 50 µM 2-APB (Fig. 4A), as expected from its ability to block CRAC channels. The peak was also greatly decreased, which is not surprising because 2-APB was originally described as a membrane permeant inhibitor of the IP_3_ receptor [32] and is well known to reduce Ca^2+^ release from stores (reviewed in [33]). Interestingly, the KCa3.1 blocker, TRAM-34, reduced the secondary phase (Ca^2+^ entry) (Fig. 4B) but did not affect the initial peak due to Ca^2+^ release from stores. The SK3 blocker, apamin, had little or no effect. To compare the overall Ca^2+^ signal, the baseline was subtracted and the area under the curve was calculated for the first 4 min after UTP was added (Fig. 4C). Compared with untreated cells, the Ca^2+^ signal was dramatically reduced by 2-APB, and to a smaller degree by omitting Ca^2+^ or adding BTP2 or TRAM-34 (but not apamin) to the bath. With Ca^2+^-free bath solution, 2-APB further reduced the peak amplitude (340/380 ratio = 2.8±0.3; not shown) and area under the curve (to 145±15), consistent with its inhibition of Ca^2+^ release from stores.

To more directly analyze the role of CRAC and KCa3.1 channels in store refilling, we applied a second UTP treatment after a 5 min recovery period (examples in Fig. 4D, E; summary in Fig. 4F). In control cells, the second Ca^2+^ rise was substantial (Fig. 4D). On average, the peak reached 67.2±4.9% of the first response (Fig. 4F), indicating that 5 min was sufficient for considerable store refilling to occur. In contrast, the peak2/peak1 ratio was markedly reduced in the absence of Ca^2+^ (to 3.9±1.8% of the control level) or in the presence of 2-APB (14.1±4.1%), BTP2 (17.5±5.4%) or TRAM-34 (17.7±4.9%) in the bath. The response with BTP2 was essentially identical to that in the Ca^2+^ free bath, as expected for normal Ca^2+^ release from stores without influx. When Ca^2+^ was omitted from the bath, the already negligible second peak was unchanged by 2-APB or BTP2 (not shown). Again, apamin had no effect. Together, these data support the hypothesis that, following P_2_Y receptor activation, Ca^2+^ influx through CRAC/Orai1 channels replenishes the depleted stores, and this is facilitated by KCa3.1 (but not SK3) channel activity.

### Evidence for a close proximity of KCa3.1 and CRAC/Orai1 channels

**Figure 5:**
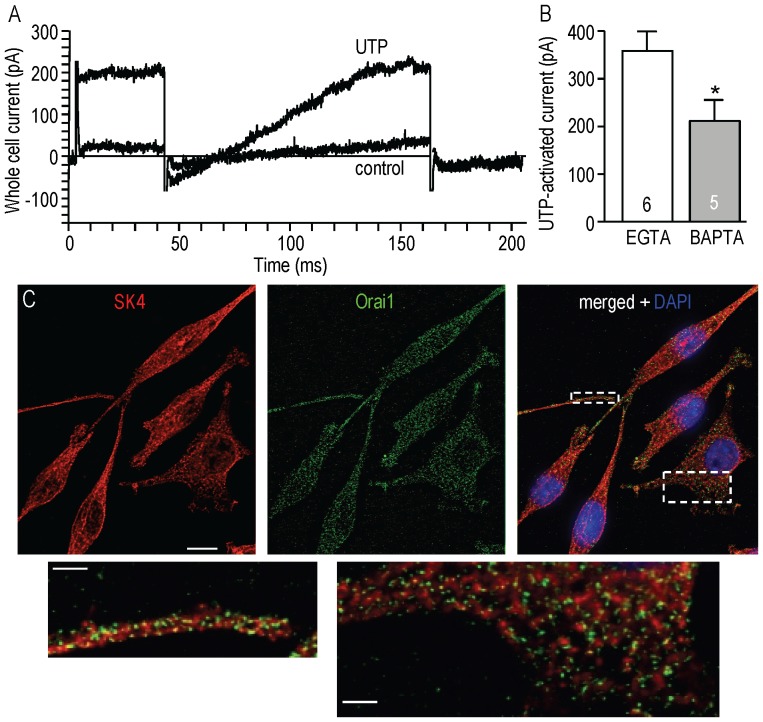
Evidence for a close proximity of KCa3.1 and CRAC/Orai1 channels. **A.** A representative whole-cell recording from an MLS-9 microglia cell with internal Ca^2+^ buffered to 120 nM using 1 mM of the fast Ca^2+^ buffer, BAPTA. The two traces show currents before (control) and after 100 µM UTP was added to the bath. The voltage protocol was the same as in Figures 1 and 2; i.e., a voltage step to +50 mV was followed by a voltage ramp from −100 to +80 mV (holding potential, −70 mV). **B.** Comparison of the KCa3.1 current amplitude at +80 mV. Intracellular Ca^2+^ was buffered to 120 nM with either 1 mM EGTA or 1 mM BAPTA (*p<0.05, Student's t-test). **C.** High-magnification, deconvolved images of MLS-9 cells show immunolabeling for KCa3.1 (red), Orai1 (green), and the nuclear marker, DAPI (blue) (scale bar  = 10 µm). Below the main panels are magnifications of the two boxed areas in the merged image (scale bars = 2 µm).

The previous results (Figs. 3, 4) show a functional coupling between Ca^2+^ influx through CRAC and KCa3.1 activation, and a reciprocal role for KCa3.1 in promoting Ca^2+^ entry and store refilling. The high global free Ca^2+^ concentration needed for KCa3.1 activation (Fig. 2) suggests that CRAC/Orai1 channels must be close to KCa3.1 channels in order for local Ca^2+^ to be high enough. A common test of proximity between a Ca^2+^ source and a responder molecule is to compare the effects of EGTA and BAPTA. While their Ca^2+^ affinities are similar at physiological pH, the binding rate constant for Ca^2+^ is 100–160 times faster for BAPTA (k_on_ = 4.5×10^8^ M^−1^s^−1^) than for EGTA (2.7×10^6^ M^−1^s^−1^) [34]. In whole-cell recordings with the slower buffer, EGTA, the amplitude of the UTP-evoked KCa3.1 current was 358±41 pA at +80 mV (Fig. 1E). Using a BAPTA-containing pipette solution with the same concentration of free Ca^2+^ (120 nM), the baseline current in standard bath was small, and UTP activated a KCa3.1 current (compare Fig. 5A with Figs. 1, 2). This means that Ca^2+^ ions entering through CRAC channels could activate some KCa3.1 channels before being chelated. However, the average KCa3.1 amplitude was significantly smaller with BAPTA (214.1±41.4 pA) than with EGTA in the pipette solution (Fig. 5B). This provides biophysical evidence for a close proximity between KCa3.1 and CRAC channels.

Next, immunocytochemistry was conducted to examine the subcellular distribution of KCa3.1 and Orai1 (CRAC) proteins in MLS-9 cells (Fig. 5C). Orai1 staining was widespread and punctate, as we recently found in primary rat microglia [11]. KCa3.1 showed widespread staining throughout the cell and at the surface, and not surprisingly, KCa3.1 and Orai1 proteins were closely associated, as seen in the two magnified regions (Fig. 5C). This is consistent with the patch-clamp results suggesting that KCa3.1 and Orai1 are physically close. Evidence of selective labeling is that negative controls were blank when the primary antibodies were omitted, and neither primary antibody revealed staining in the nucleus.

### Roles for CRAC and KCa3.1 channels in microglia migration

**Figure 6:**
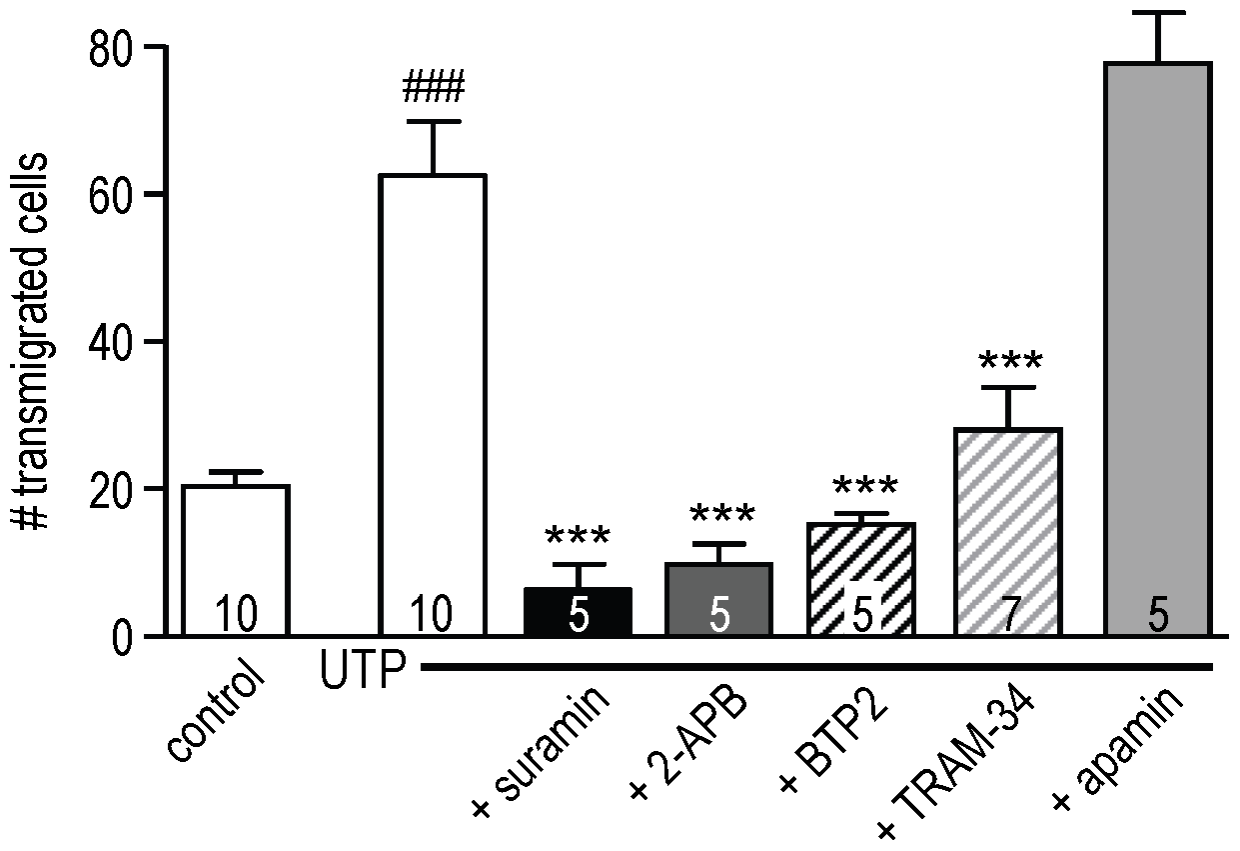
Roles for CRAC and KCa3.1 channels in microglia migration. Primary cultured rat microglia were seeded on filters with 8 µm-diameter pores and placed in the upper insert of Transwell^TM^ chambers. After 24 h, transmigration was compared without (control) or with 100 µM UTP, in the lower chamber. The following antagonists were added to the microglia-containing upper well: the P_2_Y receptor antagonist, 100 µM suramin; blockers of CRAC/Orai1 (50 µM 2-APB, 10 µM BTP2), KCa3.1 (1 µM TRAM-34) or SK1–SK3 (100 nM apamin). For each treatment, cell counts were summed from 5 random fields of view at 20× magnification. The number of separate cell cultures is indicated on each bar. Statistical differences between control and UTP-treated cells (^###^ *p*<0.001), and for UTP-treated cells with or without antagonists (***p<0.001) were determined by 1-way ANOVA with Tukey's post hoc test.

Here, we show for the first time that transmigration of primary rat microglia is increased by P2Y receptor activation by UTP (i.e., more than a 3-fold increase; Fig. 6). [Note: we always use primary microglia for functional assays.]. Transmigration and the increased migration in response to UTP was reduced 89% by suramin. Together, this pharmacology implicates the P_2_Y_2_ receptor. Most notably, transmigration was strongly inhibited by the CRAC/Orai1 channel blockers, 2-APB (reduced by 84%) and BTP2 (reduced by 76%). In addition, there was a 55% inhibition by the KCa3.1 blocker, TRAM-34. The SK1–SK3 blocker, apamin, did not reduce migration. This pharmacological profile exactly parallels that of the UTP-induced Ca^2+^ signaling and activation of KCa3.1 channels (Figs. 1, 3, 4).

## Discussion

Microglial activation is multi-faceted, with diverse Ca^2+^-dependent functions that can orchestrate the inflammatory response to CNS injury. The specificity and accurate execution of microglial functions will depend on appropriately coupling external stimuli to Ca^2+^ signals and downstream effectors. Several G-protein coupled metabotropic purinergic receptors (P_2_Y_2_, P_2_Y_4_, P_2_Y_6_, P_2_Y_12_) have been identified in microglia (reviewed in [3], [7], [35], [36]). The use of agonists and antagonists can discriminate between them, and our results implicate P_2_Y_2_. That is, the UTP concentration used (100 µM) strongly activates P_2_Y_2_ and P_2_Y_4_ receptors, but only weakly activates P_2_Y_6_ [27], [37], [38]. The response was dramatically reduced by suramin at a concentration (100 µM) that fully inhibits P_2_Y_2_, inhibits P_2_Y_ 6_ by ∼30%, and does not inhibit P_2_Y_4_ [27]. P_2_Y receptors elevate Ca^2+^ through IP_3_-mediated release from internal stores and influx through store-operated Ca^2+^ entry (SOCE). Here, UTP evoked a biphasic Ca^2+^ rise, with a rapid transient (typical of internal release) followed by a smaller plateau phase (typical of store-operated Ca^2+^ entry).

Following depletion of Ca^2+^ stores, rapid replenishment is important to prepare the cell for another stimulus; e.g., in cells with Ca^2+^ oscillations and during migration (reviewed in [39]). T lymphocyte activation is exemplary; i.e., the Ca^2+^ oscillations that are required to facilitate gene activation result from cyclical Ca^2+^ release from stores and subsequent refilling, which depends on CRAC/Orai1 (reviewed in [40]). There are multiple Ca^2+^ entry pathways in microglia (reviewed in [8], [41]), and in rat microglia, we previously found that the Ca^2+^-release-activated Ca^2+^ (CRAC/Orai1) channel is activated following store depletion (by thapsigargin) [10]. Here, we found that both the UTP-evoked Ca^2+^ plateau phase and the refilling of stores were mediated by Ca^2+^ influx through CRAC channels. Both processes were nearly abolished by omitting extracellular Ca^2+^, by the channel blocker, 2-APB and, importantly, by BTP2 at a concentration that is selective for CRAC/Orai1 channels [30], [31].

To maintain the driving force for optimal Ca^2+^ influx, a hyperpolarizing conductance is required. In microglia, depolarization reduced the Ca^2+^ rise after UTP stimulation [3], [9]. KCa3.1 channels perform this role during T cell activation ([42], [43]; reviewed in [40]) and in human macrophages [44]. Because both SK3 and KCa3.1 channels are expressed in primary rat microglia [12], [13], [45], and both currents are reliably activated (by riluzole) in MLS-9 cells [14], we had expected both to contribute in response to UTP. Surprisingly, only the KCa3.1 current was activated, and only KCa3.1 promoted the CRAC-mediated Ca^2+^ rise and refilling of stores. A reciprocal relation was seen, wherein KCa3.1 activation required Ca^2+^ influx and this was mediated by CRAC/Orai1 channels. Based on this functional coupling, we anticipated that KCa3.1 and Orai1 proteins would be in close proximity, and immunostaining showed that both were prevalent and widespread throughout MLS-9 cells. We recently reported a similar Orai1 distribution in primary rat microglia [11].

Not surprisingly, by elevating intracellular Ca^2+^, P_2_Y_2_ receptors can evoke Ca^2+^-activated K^+^ currents in some other cell types. For instance, UTP-stimulation of P_2_Y_2_ receptors activated KCa3.1 channels when exogenously co-expressed in *Xenopus* oocytes [46], and UTP can activate KCa3.1 in macrophages [44]. However, whether this involves a local trans-activation by specific Ca^2+^ channels is not known. Activation of KCa3.1 by nearby CRAC/Orai1 channels in microglia might reflect a specific coupling in non-excitable cells that do not express or use voltage-dependent Ca^2+^ channels (VDCCs). In endothelial cells, opening of TRPV4 channels evoked local Ca^2+^ “sparklets” that activated nearby small- and intermediate-conductance channels that were presumed to be KCa2.3 and KCa3.1 [47]. In some neurons, KCa2.x channels can be activated by nearby voltage-dependent Ca^2+^ channels (VDCCs), N-methyl d-aspartate (NMDA) receptors, and nicotinic acetylcholine receptors [48]. In cerebellar Purkinje cells, KCa3.1 channels interact with, and are activated by Cav3.2 VDCCs [49].

Purinergic receptors mediate numerous microglial functions. ATP is considered an important trigger for responses to acute injury, and it increases microglial motility and migration *in vivo* and *in vitro* ([6], [50], [51]; reviewed in [3], [7], [35], [36]). The focus has been on roles of P_2_X_4_ and P_2_Y_12_ receptors in chemotaxis, PLC-mediated rises in intracellular Ca^2+^, and translocation of β1 integrins [4], [5], [7]. However, P_2_X_4_ and P_2_Y_12_ receptors are not activated by UTP. We found that UTP increased migration of primary rat microglia, and the pharmacological profile clearly implicated P_2_Y_2_ receptors, just as we observed for the Ca^2+^ signaling and KCa3.1 channel activation. In P_2_Y_2_ receptor null mice, chemotaxis is impaired in neutrophils [52], monocytes and macrophages [53]. Cell migration is regulated by Ca^2+^ signals that range from transient localized ‘flickers’ to oscillations and sustained gradients [54]–[57]. In some cells, Ca^2+^ oscillations produce optimal migration [58] but there is limited information about the specific Ca^2+^-permeable channels involved. The TRPM7 channel was implicated in Ca^2+^ flicker activity during migration in a neuroblastoma cell line transfected with this channel [56]. SKF96365, a drug that blocks several Ca^2+^ permeable channels including TRPM7 and CRAC/Orai1, reduced migration of a breast tumor cell line [59]. Reduced migration was seen in neutrophils from heterozygous Orai1 knockout mice and in the HL-60 myeloid cell line after siRNA-mediated Orai1 depletion [60]. Not surprisingly, considering the roles of CRAC/Orai1 and KCa3.1 channels in UTP-evoked Ca^2+^ entry and store refilling, we found that these channels contribute to UTP-stimulated chemotaxis of primary rat microglia. We recently discovered that migrating rat microglia possess podosomes, which are tiny multi-molecular structures with dual functions in mediating cell adhesion and substrate degradation [11], [26]. Interestingly, the podosomes are enriched in Orai1 (and several Ca^2+^-dependent proteins), and blocking Ca^2+^ entry through CRAC/Orai1 channels inhibited podosome formation and migration [11]. KCa3.1 is expressed in several migratory cell types, and pharmacology implicates it in migration (reviewed in [61]–[63]. For instance, lysophosphatidic acid-stimulated chemotaxis was inhibited by charybdotoxin and clotrimazole [64], which are less selective KCa3.1 blockers than TRAM-34. Further studies will be needed to determine whether KCa3.1 and CRAC/Orai1 act in concert to facilitate UTP-stimulated microglial migration by promoting specific downstream processes, including podosome formation.

Two surprising results were that only KCa3.1 (not SK3) was activated by UTP, and channel activation was remarkably insensitive to simply elevating Ca^2+^ in the intracellular (pipette) solution. The EC_50_ was 7.6 µM Ca^2+^ and no current was seen at 2.5 µM Ca^2+^. The UTP-evoked Ca^2+^ rise peaked at 5.3 µM in the whole cell but is undoubtedly higher adjacent to open CRAC channels. While a range of EC_50_ values has been reported for activating KCa3.1 channels, they are well below 1 µM Ca^2+^ (reviewed in [65]–[67]). For instance, reports on lymphocytes indicate a threshold of about 200 nM Ca^2+^, an EC_50_ of 300–450 nM, and saturation at 1 µM [43], [68], [69]. For cloned KCa3.1 channels, the expression system might affect the Ca^2+^ sensitivity; e.g., an EC_50_ of 95 nM Ca^2+^ was seen in CHO cells [70] versus 700 nM in *Xenopus* oocytes [71]. The biological basis for these differences is unknown. While we do not know the reasons for the very high EC_50_ for activating KCa3.1 or the failure to activate SK3 channels in the present study, it is worth considering the known mechanism for activating these channels.

SK and KCa3.1 channels are tetramers and are activated by Ca^2+^ binding to calmodulin, which is bound to the CaM-binding domain (CaMBD) in the proximal C-terminus of each channel monomer [43], [71], [72]. Each CaM molecule has four E-F hands that can potentially bind Ca^2+^; two each in the C- and N-lobes, which are connected by a flexible linker region [73]. Only one E-F hand in the N-lobe is apparently required for the Ca^2+^-dependent gating of SK channels and this produces a Hill coefficient of ∼4 in the Ca^2+^-dose-response curve [73], [74]. For KCa3.1, Hill coefficients of 3.9 [68], 3.2 [70], and 4.6 (present study) have been reported. Thus, it is unlikely that the low Ca^2+^ sensitivity in this study reflects fewer functional Ca^2+^ binding sites. The selective activation of KCa3.1 but not SK3 is intriguing because we recently found that the neuroprotective drug, riluzole, reliably activates both channels in MLS-9 cells [14]. For heterologously expressed channels, riluzole shifts the Ca^2+^ dependence to the left, thereby increasing the probability of channel opening at lower Ca^2+^ levels. Riluzole reduced the EC_50_ from 470 to 112 nM Ca^2+^ for expressed rat SK2 [15], activated rat SK3 at 100 nM intracellular Ca^2+^ [75], and increased the current amplitude by 30-fold at 250 nM Ca^2+^ for human SK3 and KCa3.1 channels [76]. The mechanism for riluzole activation of SK3 and KCa3.1 must be different in MLS-9 cells because micromolar levels of Ca^2+^ alone did not activate the currents (present study), and riluzole elevated Ca^2+^ only to 200 nM [14]. One possibility is that P_2_Y_2_ receptor activation affects an unidentified accessory molecule that aids in channel activation.

For SK2 and SK3 channels, Ca^2+^ sensitivity is affected by CaM phosphorylation by CK2 protein kinase and PP2A protein phosphatase, which bind to the channels [77], [78]. Thus, it is worth considering what is known about factors that regulate the Ca^2+^-sensitivity of KCa3.1 channels. While some studies show modulation by ATP in the intracellular solution, there is some conflicting data. The EC_50_ for channel activation in transfected oocytes was 490 nM Ca^2+^ without ATP and 320 nM with ATP [79]. For endogenous KCa3.1 channels in rat submandibular acinar cells, the K_d_ was 1.35 µM without ATP and 0.66 µM with ATP [80]. These small ATP effects cannot account for the high K_d_ (7.6 µM Ca^2+^ with ATP always present) in our study of KCa3.1 in microglia. KCa3.1 regulation might be cell-type specific. For instance, our early study suggested that CaMKII regulates the endogenous KCa3.1 channel in T cells but not the expressed channel in CHO cells [43]. Two studies showed that activation of KCa3.1 by hydrolyzable ATP analogues occurred with no change in Ca^2+^ sensitivity [81] and that channel activation was due to PKA in *Xenopus* oocytes (transfected) and in the T84 cell line (non-transfected) but not in transfected HEK cells [82]. However, another study of KCa3.1-transfected oocytes concluded that PKA inhibits the channels, and is independent of mechanisms controlling Ca^2+^ sensitivity [83]. While KCa3.1 channels interact with, and are regulated by some kinases and phosphatases (for an excellent recent review, see [84]), there is no evidence that they can modulate the Ca^2+^ dependence to account for the low sensitivity of the microglial KCa3.1 channel. AMP-activated protein kinase (AMPK) inhibits the current and directly binds to the distal C-terminus of the KCa3.1 protein [85]. The reported regulation by PKC is likely indirect, and was not affected by mutating the PKC consensus sites [86]. Recent discoveries concerning KCa3.1 regulation have begun to identify interacting proteins. The lipid PI_3_P phosphatase, myotubularin-related protein 6 (MTMR6), interacts with KCa3.1 and inhibits its function by dephosphorylating PI_3_P near the channel [87]. The histidine kinase, nucleoside diphosphate kinase B (NDPK-B) binds to and activates KCa3.1 [88]; an effect that is reversed by protein histidine phosphatase (PHPT-1) [89].

A recent study found that CaM-CaMBD interactions change the CaM conformation and increase its Ca^2+^ affinity [73]. While numerous SK1–SK3 splice variants have been found (some differing in the C-terminus that contains the CaMBD) (reviewed in [90]), very little is known about their Ca^2+^ sensitivities. Most relevant is the recent discovery of an SK2 variant with three extra amino acids in the CaMBD [73]. It has a reduced Ca^2+^ sensitivity, with an EC_50_ of 1 µM rather than ∼300 nM, but no change in the Hill coefficient. Three KCa3.1 variants have been found in rat colon: KCNN4a, KCNN4b and KCNN4c encode proteins of 425, 424, and 395 amino acids, respectively [91]. KCNN4b lacks a glutamine at position 415, and KCNN4c lacks the S2 transmembrane segment, but Ca^2+^ sensitivities were not examined. A dominant-negative variant found in lymphoid tissues lacks the N-terminus, and because it suppresses normal membrane KCa3.1 expression [92], is unlikely to account for the present results. Further studies will be needed to address whether microglia express a KCa3.1 channel splice variant that is less sensitive to Ca^2+^.

This work has broader implications because KCa3.1 channels are expressed in numerous cell types (mainly non-excitable), and have been implicated in a range of cell functions, as extensively reviewed in recent years [65], [84], [93]–[98]. For instance, initially described from studies of red blood cell volume regulation, KCa3.1 is now known to regulate activation of subsets of T lymphocytes, mediate salt and water transport across epithelia, regulate endothelial cell contributions to vascular tone, and modulate cell proliferation and differentiation of several cell types. There is less known about KCa3.1 in the CNS and it was initially thought to be absent from the brain [99]. KCa3.1 is present in microglia [45], [100], and we showed it is involved in p38 MAPK activation and subsequent superoxide and nitric oxide production [12], [45]. Blocking KCa3.1 with TRAM-34 reduced the ability of microglia to kill neurons *in vitro* and decreased retinal ganglion cell degeneration *in vivo* [12]. KCa3.1 is being considered as a clinical target for multiple diseases, from sickle cell anemia to inflammation, gastrointestinal disorders, heart disease, multiple sclerosis and stroke. Thus, there is interest in cell-specific mechanisms that control channel activation, Ca^2+^ sensitivity, and coupling to specific receptors and channels.
